# Does a diagnosis of depression influence observer ratings of pain severity? The mediating role of causal attributions of pain and pain genuineness

**DOI:** 10.1177/20494637231206541

**Published:** 2023-10-18

**Authors:** Kara Turcotte, Susan Holtzman

**Affiliations:** 1 Department of Nursing, 6221 Western University, Kelowna, ON, Canada; 2 Department of Psychology, 97950 University of British Columbia - Okanagan, Kelowna, BC, Canada

**Keywords:** chronic pain, musculoskeletal pain, pain, pain perception, pain measurement

## Abstract

Researchers have been increasingly investigating observer and patient characteristics that may influence the assessment of pain in others. While rates of psychiatric conditions are high in chronic pain populations, surprisingly little attention has been given to if (and why) a comorbid psychiatric diagnosis may influence the estimation of pain in others. Using an experimental vignette paradigm, the current study examined whether a diagnostic label of major depressive disorder (MDD) would impact observer pain estimates of a woman with chronic pain, and whether causal attributions of pain and pain genuineness might help explain these effects. Participants (*n* = 188) were given a vignette describing a female patient with chronic pain (who either had MDD or no mental health concerns), viewed a brief video clip of the patient, and then were asked to provide a variety of ratings about the woman’s pain. Results of a serial multiple mediation analysis revealed that participants in the MDD condition made greater psychological attributions for the woman’s pain, which was associated with lower perceptions of pain genuineness, which was then associated with lower estimates of pain intensity. These findings suggest that a diagnosis of depression may indirectly influence observer estimates of another person’s pain by heightening psychological attributions of pain, and making their pain seem less genuine. Further research is needed to elucidate the complex processes underlying pain estimation, including patient and observer characteristics, biases, and heuristics, in order to improve quality of care for those living with persistent pain.’

## Introduction

Accurately assessing the pain of others is a challenging task. Whether the observer is a health care provider, family member, or stranger, estimates of another person’s pain are typically inaccurate.^1–5^ The biopsychosocial communication model of pain suggests that pain is an internal subjective experience that cannot be observed directly, but rather, it must be inferred via verbal and nonverbal behavior.^6^ Within this framework, researchers have been increasingly investigating observer and patient characteristics that may influence the accuracy of pain assessment.^7–9^

Although rates of psychiatric conditions are high in chronic pain populations,^10^ suprisingly little attention has been given to if (and why) a comorbid psychiatric diagnosis may influence the estimation of pain in others. Depression is the most common psychiatric comorbidity among those with chronic pain.^11,12^ It is estimated that between 5% and 85% of patients with a pain condition have depression and approximately two-thirds of patients with major depressive disorder (MDD) have one or more pain complaints.^13^ Pain with comorbid depression has been linked with an array of adverse outcomes, ranging from more intense pain, greater impairments in social functioning, and diminished quality of life, compared to those without depression.^10^ There are many reasons why this may be the case, including overlapping neurological pathways^14^ and maladaptive coping mechanisms like pain catastrophizing.^15^ Worse pain-related outcomes among those with comorbid depression may also be due to problems originating at the stage of pain assessment.

Vignette studies have shown that formal and informal care providers may underestimate pain in individuals with psychiatric comorbidities.^16,17^ Patients with chronic pain who are believed to be depressed may also be rated as less trustworthy^18^ and vignette research has shown that (female) patients who are seen as less trustworthy are perceived to have less pain and to be more likely to exaggerate their pain.^19^ Observers may also be more likely to attribute pain to psychological causes (rather than physiological causes) when patients are believed to suffer from a psychiatric condition.^20,21^ From the patient perspective, those living with pain conditions with particularly high rates of depression and mood disturbance (e.g., fibromyalgia) commonly report experiences of invalidation from support providers, such as disbelieving and discounting pain, and a general lack of understanding (e.g., Refs. ^22,23^) Past experimental research has also shown that when a lack of medical explanation for pain is combined with information that psychosocial factors are at play, an observer’s sympathy and perceptions of genuineness may decline.^24^ This latter finding is particularly concerning, given that a high proportion of pain symptoms that present in primary care settings have no clear medical explanation.^25–27^

A need for a focused investigation into the impact of psychiatric comorbidity on pain assessment, specifically in women, is well-supported by the research literature. Rates of both chronic pain and depression are substantially higher among women, compared to men.^28,29^ Women’s pain is also more likely to be underestimated^9,30,31^ (for an exception, see Ref. ^32^) to be undertreated,^33^ and to be treated with psychiatric medication.^34^ A review of clinical and experimental pain research has suggested that psychosocial factors may be at least partly responsible for these gender differences and inequities.^31^ Since rates of depression are substantially higher among women with chronic pain,^28^ it stands to reason that depressive comorbidity could be at least partly responsible for some of the disparities documented in past research.

Using an experimental vignette paradigm, the first aim of the current study was to examine whether a diagnostic label of major depressive disorder (MDD) would impact observer pain estimates in a female patient with chronic pain. It was hypothesized that participants assigned to the MDD label condition would rate the patient as experiencing less pain, compared to those in the no MDD label condition. The second aim was to examine potential mediators of the association between MDD and ratings of pain intensity. A serial multiple mediation model was employed to test two hypothesized pathways. First, a comorbid label of MDD was expected to be associated with higher psychological pain attributions, which would then be associated with lower ratings of pain genuineness, which in turn would lead to lower pain ratings. Second, an MDD diagnosis was expected to be associated with lower physical pain attributions, which would be associated with lower ratings of pain genuineness, and lower pain ratings. This study is among the few studies to date that have experimentally tested a mediation model to explain why women with comorbid depression and chronic pain may be at increased risk of inadequate pain management.

## Methods

### Participant sample and recruitment

Participants (*n* = 188) were predominantly female (77.1%) and identified as Caucasian (77%), ranging from 18 to 43 years of age (M = 19.92, SD = 1.95). Participants were recruited through the psychology research subject pool at a mid-sized Canadian university and received course credit for completing the study. To be eligible, participants were required to be at least 18 years of age, and fluent in English.

### Procedure

This online study was part of a larger study investigating psychosocial factors that influence the perception of pain in others. As such, only the variables pertinent to the present research questions will be discussed below. The procedure was conducted using Qualtrics, an Internet-based survey platform (Qualtrics, Provo, UT).

### Vignette

After providing informed consent, participants were randomized (using an embedded function in Qualtrics) to read one of two vignettes, which both contained a fabricated medical case history of a middle-aged woman experiencing explained pain (i.e., persistent shoulder pain after falling off a ladder). We chose “medically explained pain” (pain related to a specific injury) in our vignette because past research has shown a detrimental impact of so-called “medically unexplained pain” on observer judgments of pain (e.g., Refs. ^25,35^), which would have made it more difficult to tease apart the role of psychiatric diagnosis. The vignettes contained information typically displayed in a medical case history, such as chief health complaint, history of present illness, past medical history, and psychiatric history. The two vignettes contained the same information, with the exception that participants in Condition 1 were informed that the patient “was recently diagnosed with a mood disorder called major depression” and participants in Condition 2 were told that the patient “does not suffer from any past or present mental health problems.”

### Video

Next, participants watched a brief 50-second silent video clip of a middle-aged Caucasian female with persistent pain rotating her injured and uninjured arms. This video clip was taken from the UNBC-McMaster shoulder pain expression archive, with permission from the Pittsburgh University’s Affect Analysis Group. This archive contains information on 25 patients with chronic shoulder pain who allowed their data to be used in future research on pain assessment. The details of the UNBC-McMaster Shoulder Pain Expression archive are provided elsewhere.^36,37^ Patients in this database used a 10 cm Visual Analog Scale (VAS), anchored at each end with the words “No pain” and “Pain as bad as it could be,” to self-report the amount of pain they experienced during each rotation trial. The patient used for this study self-reported a maximum pain intensity of 7.0 on the VAS during the active movement of the affected arm and a moderate amount of pain across the length of the video clip (average VAS 4.3).

### Post-vignette questionnaires

In order to determine whether participants were attending to the specific details contained in the vignette and video clip, participants were first asked several multiple choice questions, including whether or not the patient they viewed had been diagnosed with a mental illness. Participants were then asked to rate the patient’s pain intensity using a 10cm Visual Analog Scale (VAS) anchored with the words “No pain” and “Pain as bad as it could be.” Next, based on previously published vignette research,^38^ participants were asked to provide their judgments regarding the patient’s psychological distress (“The individual is experiencing psychological distress”), pain genuineness (“The pain is genuine”), psychological attributions (“The pain is attributable to psychological factors”), and physical attributions (“The pain is attributable to physical factors”). Each item was assessed on a 7-point Likert scale ranging from “strongly disagree” to “strongly agree.” Lastly, participants were asked to complete a series of standardized self-report questionnaires, including a measure of demographic information assessing their age, gender, and ethnicity.

### Statistical analysis plan

An a priori power analysis was conducted using G*Power.^39^ It was determined that for a medium size effect, 107 participants would be required. Preliminary analyses involved a manipulation and randomization check. The manipulation check was conducted to ensure participants had been attentive to the information provided in the medical case history vignette, and in particular, to the presence or absence of major depression. We also compared participants’ estimates of the patient’s psychological distress using an independent samples *t* test, expecting that participants in the MDD condition would rate the patient as being in more distress than the participants in the no MDD condition. As a randomization check, independent samples *t*-tests were conducted to test for any differences in the demographic characteristics of the participants assigned to the two conditions. A descriptive analysis was also conducted on the demographic and key study variables. Given previous research suggesting that observer gender can impact pain judgments,^38^ Welch’s t-tests were also conducted to detect possible differences in ratings of pain intensity, genuineness, causal attributions, based on the participants’ gender.

The first study aim, which addressed the question of whether pain estimates differ between the MDD and no MDD conditions, was evaluated in two ways. First, independent samples t-tests examined differences in pain intensity ratings between the MDD and no MDD conditions. Second, the accuracy of pain estimates between the MDD and no MDD conditions was examined, to determine whether there were differences in the likelihood to under-, over-, or accurately estimate the patient’s pain based on study condition. Patient-participant concordance in pain ratings was operationalized using the procedure devised by Ref. ^40^. Difference scores were first calculated between participants’ estimates of the patient’s pain and the patient’s self-reported pain (VAS = 4.3). Participant pain estimates that were within one centimeter point of the patient’s self-report (i.e., either above or below) were defined as being concordant. Participants who rated the patient’s pain as more than one centimeter point lower than the patient’s self-reported pain comprised the underestimation group and participants who rated the pain as more than one centimeter point higher than the patient’s self-reported pain comprised the overestimation group. A Chi-square test was then conducted to determine whether rates of overestimation, underestimation, or accurate assessments differed between the MDD and no MDD conditions.

The second study aim involved a test of the indirect effects of a label of MDD on pain intensity, through pain attributions and pain genuineness. A serial multiple mediation model was tested using the PROCESS macro Model 6, a software add-on for SPSS, which uses an ordinary least squares regression-based path analytic framework for estimating direct and indirect effects (Process 3.5^41^). Recent methodological research has demonstrated that a significant indirect effect can be found in the absence of a total effect, supporting the use of mediation analysis even when a significant association is not found between X (predictor) and Y (outcome; for example, Refs. ^42,43^). In the mediation model, indirect effects were calculated using bias-corrected bootstrapping, a non-parametric approach, with 10,000 resamples. Indirect effects are classified as significant if the 95% confidence interval does not include zero. All statistical analyses were conducted using SPSS (Version 27).

## Results

### Preliminary analyses

In total, 88 of 102 (86%) of the participants in condition one (MDD label), and 100 of 110 (90%) of the participants in condition two (no MDD label) passed the manipulation check. Participants in each of the conditions were equally likely to pass the manipulation check, χ^2^ (1, *n =* 211) = 1.53, *p* = .28. Those who failed the manipulation check (*n* = 24) were excluded from the analyses. As expected, estimates of psychological distress were significantly higher among those assigned to the MDD condition (M = 4.01, SD = 1.49), compared to the no MDD condition (M = 3.09, SD = 1.44), *t* (186) = −4.30, *p* < .001. No significant differences between the MDD and no MDD conditions emerged with respect to participant age, gender, or ethnicity (all *p*’s > 0.11), suggesting that random assignment was successful. Estimates of the patient’s pain intensity were not significantly different between male (M = 4.40, SD = 1.93) and female (M = 4.65, SD = 1.87) participants, *t* (186) = −0.75, *p* = .453. A Welch’*t* test found that male and female participants did not differ significantly in their psychological or physical attributions of the patient’s pain, or the perceived genuineness of the patient’s pain (all *p’*s > 0.273).

Means and standard deviations for the study variables are presented in Table 1 (across the study sample and for each study condition). Effect sizes are presented in Table 1 and can be interpreted as small (Cohen’s *d* > 0.20), medium (Cohen’s *d* > 0.50), and large (Cohen’s *d* > 0.80^44^). A label of MDD had a small-sized effect on ratings of pain genuineness (*d =* 0.23) and medium-sized effect on psychological attributions for the cause of the patient’s pain (*d* = 0.64). Across the study sample, higher ratings of physical attributions for pain and pain genuineness were correlated with higher pain estimates (*r* = 0.23 and *r =* 0.39, *p*’s < .01, respectively). Higher psychological attributions were correlated with lower ratings of pain genuineness (*r* = −0.23, *p* < .01), but not with physical attributions (*r* = −0.08, *p* = .29) or estimates of pain intensity (*r* = 0.07, *p* = .35).

**Table 1.** Descriptive results of study outcome and mediator variables.

Variables		Overall	No MDD condition	MDD condition	*t* test	Cohen’s d
		M(SD)	M(SD)	M(SD)		
		*n* = 188	*n* = 100	*n* = 88		
Outcome	Pain intensity	4.59 (1.88)	4.59 (2.00)	4.60 (1.75)	−0.04	.01
Mediators	Pain genuineness	4.85 (1.30)	4.99 (1.28)	4.69 (1.32)	1.56	.23
	Psychological attribution	3.36 (1.65)	2.89 (1.50)	3.90 (1.66)	−4.38***	.64
	Physical attribution	5.37 (1.20)	5.41 (1.18)	5.32 (1.22)	.52	.08

*Note.* M = Mean, *SD* = Standard Deviation.*t* test = Independent samples *t* test, *p < .05 **p < .01 ***p < .001. Possible scale of 1–7/0–10. No MDD Condition dummy coded as 0, MDD condition dummy coded as 1.

### Study aim 1: The impact of a label of MDD on observer ratings of pain intensity

Between the MDD and no MDD conditions, there was no significant difference in participants’ estimates of the patient’s pain intensity, *t* (186) = −0.04, *p* = .969, Cohen’s *d* = 0.01 (see Table 1). The accuracy of pain estimates also did not differ between the MDD and no MDD conditions, χ^2^ (2, *n* = 188) = 59.46, *p* = .422. Overall, across the two conditions, 43.61% of participants were classified as having accurately estimating the woman’s pain, whereas 32.45% overestimated, and 23.94% underestimated the pain.

### Study aim 2: The mediating role of pain genuineness and pain attributions

**Figure 1.**
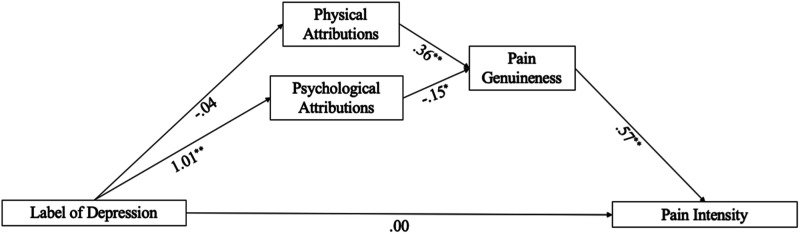
Serial multiple mediation model. *Note.* Only the hypothesized effects are presented. ^*^p < .05 ^**^p < .01.

Results of the serial multiple mediation analysis are presented in Table 2 and Figure 1. Physical attributions for pain did not mediate the relationship between the label of MDD and pain intensity (β = −.01, 95% CI −0.08, 0.08). An indirect effect was found whereby psychological attributions mediated the relationship between the label of MDD and pain intensity ratings (β = .19, 95% CI 0.02, 0.43). A significant association between physical pain attributions and higher perceptions of pain genuineness was found (β = 0.36, 95% CI 0.17, 0.55), as well as psychological pain attributions and lower perceptions of pain genuineness (β = −0.15, 95% CI −0.28, −0.03). A significant association was also found between genuineness and pain intensity, (β = 0.57, 95% CI 0.31, 0.83).

**Table 2.** Total and indirect effects of serial multiple mediation model for physical attributions, psychological attributions, and pain genuineness mediating the association between MDD and pain intensity.

Pathway	β	SE	95% CI	
			Lower	Upper
Direct effect	0.002	0.263	−0.517	0.522
Total indirect	0.009	0.156	−0.288	0.326
MDD→PsychA→PI	0.194	0.106	0.017	0.434
MDD→PhysA→PI	−0.006	0.037	−0.078	0.08
MDD→PG→PI	−0.064	0.11	−0.295	0.142
MDD→PsychA→PG→PI	−0.088	0.05	−0.205	−0.013
MDD→PhysA→PG→PI	−0.008	0.038	−0.078	0.079

*Note.* Only the hypothesized indirect effects are presented. CI, Confident Interval; MDD, Label of major depressive disorder; PhysA, Physical attributions; PsychA, Psychological attributions; PI, Pain Intensity; PG, Pain Genuineness.

The relationship between the MDD label and pain intensity was mediated by psychological attributions and assessments of pain genuineness, as indicated by the significant indirect effect (β = −.09, 95% CI −0.21, −0.01). Specifically, a label of MDD was associated with higher psychological attributions for pain, which was related to lower perceptions of pain genuineness, which in turn was related to lower estimates of pain intensity. However, contrary to study hypotheses, the indirect effect via physical attributions was not significant. The direct (β = 0.002, 95% CI −0.52, 0.52) and total indirect (β = 0.009, 95% CI −0.29, 0.33) effects for the serial multiple mediation model were also non-significant.

## Discussion

The current experimental vignette study addressed multiple calls in the literature to investigate psychosocial factors that may influence the perception of pain in others.^6,25,45,46^ This study also addressed the need to better understand how a woman’s mental health, specifically depression, may impact observer ratings of pain.^45^ These are critical areas of research given what is known about the potential impact that observer judgments about pain can have on patient’s wellbeing in both formal and informal support settings^47^ (Creamens-Smith et al., 2003; Ref. ^48^). Our results showed that for a woman with chronic pain, a diagnosis of major depressive disorder was associated with higher psychological attributions for that pain, which was related to lower ratings of pain genuineness, which in turn were related to lower estimates of pain intensity.

Findings from the current study help to clarify the limited and mixed findings regarding the impact of comorbid psychiatric issues on estimates of other people’s pain. For example, in one vignette study, the presence of psychosocial distress (operationalized as “depression and relational difficulties”) was found to lead observers to rate a patient as being in less pain.^17^ However, in another vignette study, a sample of health care students were told that a patient had received a referral to a psychologist for their pain, and this information did not influence pain judgments.^49^ Similarly, there was no direct relationship between a diagnosis of MDD and estimates of the patient’s pain in the current study. It was only when we took into account the mediating role of causal attributions of pain and perceptions of pain genuineness that a small but significant indirect path emerged. Indeed, past research has shown that pain may be perceived as less genuine in the presence of psychiatric comorbidity and that less trust and perceived genuineness appears to lower estimates of people’s pain.^19,50^ Taken together, these findings highlight the complex role of mental health, attributions, and perceived genuineness in the judgments that people make about others’ pain.

Contrary to study hypotheses, a diagnostic label of MDD did not predict lower physical attributions of pain, and the total indirect effect of the model was not significant. This could be related to the decision to use “medically explained pain” in the vignette (i.e., pain that was the direct result of a specific injury). The mean levels of physical pain attributions were high across the two study conditions, and it is possible that this may have overpowered the effects of the diagnostic label of MDD. This idea is in line with recent research demonstrating that medical evidence for pain can influence judgments of patients with chronic pain in lay populations, and specifically that observers are more skeptical of chronic pain not supported by medical evidence.^50^

Across the two experimental conditions, the accuracy of pain estimates was poor, with only 43.61% concordance (32% overestimated and 24% underestimated). This is consistent with previous research that highlights the prevalence of misestimating pain in others.^3^ Findings from the current study indicate that individuals draw on a range of information to make judgments about someone else’s pain that go far beyond any available medical information and physical expressions of pain, and these may lead to over- or under-estimations of people’s pain.

### Limitations and future directions

One limitation of the current research is that participants were recruited from a psychology research subject pool and thereby might have been primed to consider psychological and social factors in the assessment of pain in others. Further, the participants were primarily young, educated, female, and identified as Caucasian. It is noteworthy that worldwide, women are the predominant providers of informal care^51^ and form the majority of health care workers.^52^ Nonetheless, future research with more diverse participants is required (e.g., age, socioeconomic status, ethnic/cultural background), as well as individuals who are more routinely involved in the assessment of pain (e.g., relatives of individuals with chronic pain, health care professionals).

Vignette research provides a number of methodological advantages, particularly the ability to intensively and systematically investigate areas that would be unethical to experimentally manipulate.^53^ However, shortcomings related to the generalizability and ecological validity of vignette research are important to note. More research, such as observational research of patient-provider and patient-caregiver interactions, would be valuable in bridging the gap between experimental research methods and clinical situations. There are also limitations to our decision to use a diagnostic label of MDD to test how depression may impact observer perceptions of pain. While depression is the most common psychiatric comorbidity among those with chronic pain,^11,12^ individuals in everyday situations may be primed about the presence of depression in a more indirect, but meaningful manner. For example, observable symptoms of depression such as sadness, lack of interest and motivation, and fatigue may have a more potent impact on observer estimates of pain intensity. We also used an example of “medically explained pain” in our vignette, which may have increased overall perceptions of pain genuineness and physical attributions for the person’s pain. Future research should investigate the impact of a label of depression in the context of pain that does not have a clear underlying injury or disease process,^35^ where the processes observed here may be even more pronounced. To that end, recent research has found that individuals with chronic pain conditions with less diagnostic certainty (e.g., fibromyalgia) report more stigma compared to those with pain conditions with greater diagnostic certainty (e.g., rheumatoid arthritis).^54^

Additional avenues for future research include greater attention towards racially/ethnically minoritized individuals (who are often at greater risk of both mental health and chronic pain conditions)^55^ as well as other stigmatized mental health conditions, including psychosis and personality disorders.^56,57^ An intersectionality framework would helpful in better understanding the manner in which diverse social identities (e.g., gender, sexuality, race/ethnicity, and socioeconomic status) interact in complex ways to influence how pain is perceived by others.^58,59^ Finally, as only a female patient was used in the vignette paradigm, the extent to which our findings are unique to female patients remains unclear. Greater diversity with respect to sex and gender is needed in study paradigms to assess related biases in pain judgments (e.g., Ref.^60^), as these variables can contribute to disparities in health care. Future research investigating the impact of psychiatric comorbidity could also measure gender role expectations in the pain experience,^61,62^ including gender-related stereotypic attributions of pain sensitivity, pain endurance, and willingness to report pain.

### Conclusions

Chronic pain conditions are diagnostically complex, particularly when the pain is medically unexplained and/or when there are psychosocial factors that may be implicated in the cause of pain.^9,46,50,63–65^ In the current research, no direct effects of a diagnostic label of depression were found on observer estimates of patient pain. However, a label of depression did appear to impact observers’ beliefs about the cause of patients’ pain, and this subsequently impacted estimates of pain genuineness and pain intensity. Fully elucidating the processes underlying pain estimation, including patient and provider characteristics, biases, and heuristics, are critical to understanding and improving the assessment of pain.^3,66^
